# Simulation-based training for teaching infection prevention: from realistic to virtual environments

**DOI:** 10.1590/0034-7167-2024-0288

**Published:** 2025-09-01

**Authors:** Raissa Silva Souza, Dárlinton Barbosa Feres Carvalho, Adriana Maria da Silva Félix, Ana Angélica Lima Dias

**Affiliations:** I Universidade Federal de São João del-Rei. Divinópolis, Minas Gerais, Brazil; II Universidade de São Paulo. São Paulo, São Paulo, Brazil

**Keywords:** Technology, Simulation Training, Clinical Reasoning, Nursing, Infections, Tecnología, Entrenamiento Simulado, Razonamiento Clínico, Enfermería, Infección

## Abstract

**Objectives:**

to describe the process of transitioning from a realistic simulation to a computer-based simulation focused on bloodstream infection prevention.

**Methods:**

this report presents the development of a technology-based solution (a 3D virtual environment simulator) to address a specific problem: expanding the reach of an existing realistic simulation. The epistemological and methodological approach of Design Science Research guided the process.

**Results:**

we developed a computer-based simulation scenario using the Unity 3D platform. A panel of experts in the field positively evaluated the resulting artifact, indicating that it possesses quality, rest on valid theoretical foundations, and effectively addresses its intended problem.

**Final Considerations:**

the transition process described led to the development of a computer-based simulation that promotes autonomous, interactive, and engaging learning. However, it serves as a complementary technological tool rather than a substitute for laboratory-based training and clinical experiences.

## RESUMEN

**Objetivos:**

describir el proceso de transición de una simulación realista a una basada en computadora sobre la prevención de infecciones del torrente sanguíneo.

**Métodos:**

este relato presenta una solución tecnológica (simulador en entorno virtual 3D) para un problema (ampliar el alcance de una simulación realista existente), contextualmente situado, utilizando el enfoque epistemológico-metodológico Design Science Research.

**Resultados:**

en la plataforma Unity 3D, se desarrolló un artefacto (escenario de simulación computarizada), evaluado positivamente por un panel de expertos. Se concluyó que el artefacto posee calidad, se basa en fundamentos teóricos válidos y puede resolver el problema al que se destina.

**Conclusiones:**

el proceso de transición generó una simulación computarizada que favorece el aprendizaje autónomo, lúdico e interactivo. No obstante, es una alternativa tecnológica complementaria a las prácticas de laboratorio y las experiencias clínicas.

## INTRODUCTION

The current educational landscape emphasizes teaching methodologies, making them as important as learning content. Various methods and strategies, guided by distinct theoretical and pedagogical orientations, have been employed to address the challenges encountered in real-world practice settings^(1)^.

In the healthcare field, there is a preference for methodologies that enable experimentation, reflection, and active knowledge construction by learners, a trend observed both in official documents and scientific publications^(1)^. Specifically in nursing education, clinical simulation has been recognized as a pedagogical strategy capable of creating conditions for knowledge acquisition and retention, as well as fostering the development of essential technical and non-technical competencies, including psychomotor, cognitive, and attitudinal skills required for professional practice^(2)^.

Regarding teaching best practices for preventing healthcare-associated infections (HAIs), the literature^(3,4)^ already presents realistic clinical simulation scenarios that allow students and healthcare professionals to review and reframe their practices, making them more effective and safer. However, implementing these scenarios across different settings has been limited due to environmental inadequacies, resource scarcity at various levels^(4)^, and insufficiently trained personnel. In this context, shifting toward digital solutions through the virtualization of realistic simulation appears to be a promising alternative to overcome these limitations.

Computer-based simulation is an educational resource that employs computational technologies to create interactive virtual learning environments. Within these environments, participants engage in complete patient care scenarios, experiencing the entire process—from cognitive engagement and reflective analysis to decision-making and intervention execution—in a controlled and safe environment for both participants and patients^(5)^.

The potential benefits of computer-based simulation in education include cost reduction, lower risk and anxiety levels, large-scale training feasibility, reproducibility, elimination of the need for physical spaces, and reduced reliance on material and highly specialized human resources^(6)^. Additionally, this strategy has proven effective in nursing education, as it enhances students’ and professionals’ critical thinking and clinical reasoning skills, enabling them to provide safe patient care, make accurate inferences, and base decisions on evidence. Nevertheless, to fully realize these potential benefits, research and technological development must be directed toward creating innovations tailored to the intended educational objectives.

## OBJECTIVES

To describe the process of transitioning from a realistic simulation to a computer-based simulation focused on bloodstream infection (BSI) prevention.

## METHODS

This report presents the development of a technology-based solution—a 3D virtual environment simulator—designed to address a specific problem: expanding the reach of an existing realistic simulation. The epistemological and methodological approach of Design Science Research (DSR) guided the process.

DSR is a technology-driven problem-solving paradigm that focuses on creating artifacts to address relevant, complex, and contextually grounded challenges^(7)^. This approach entails both a rigorous scientific investigation into the artifact development process and an evaluation of its suitability and relevance in problem-solving^(7)^.

**Figure 1:**
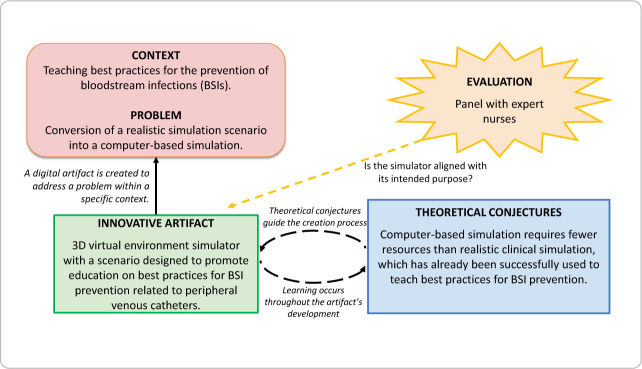
Methodological framework considered in this study

The core methodological framework applied in this study, as outlined by DSR, is represented in Figure 1, which presents a synthesized model inspired by the work of Pimentel, Filippo, and Santoro^(7)^.

The context of this study involves teaching nursing students and professionals the best practices for bloodstream infection (BSI) prevention related to peripheral venous catheters (PVCs). This target audience had access to a validated clinical scenario designed by subject matter experts for hybrid realistic simulation, combining simulated patients and a low-fidelity simulator for intravenous injections^(7)^. One approach to expanding the reach of this simulation was to transition it to a virtual environment (the identified problem) through the development of an artifact (a 3D virtual environment simulator).

**Figure 2:**
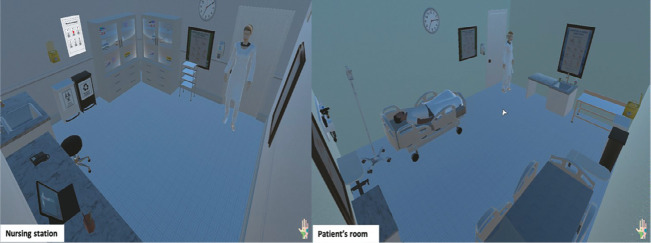
Representation of the nursing station (left) and the patient’s room (right) in the clinical scenario, modeled on the Unity 3D computational platform

We conducted the study in accordance with the following DSR stages^(8)^: (1) Awareness – identification and definition of the problem to be solved, as outlined in Figure 1, (2) Suggestion – listing potential artifact solutions and selecting the most suitable one for development (a three-dimensional virtual environment simulator), (3) Development – execution of the artifact’s construction process, (4) Iterative evaluation – assessment of the artifact by nurse specialists in the thematic area, considering the theoretical foundations of its development and its performance within the intended simulation environment, (5) Conclusion – preparation of the present manuscript to formalize the development process and document the findings, and (6) Communication.

Recognizing the importance of iterative technological development for complex application domains^(9)^, the team created a low-fidelity prototype for the evaluation phase. This prototype aimed to demonstrate the design decisions made by the developers and present the artifact’s performance, serving as a final validation step to confirm its adequacy.

Considering that using low-fidelity prototypes in technology evaluation processes remains uncommon in nursing, a virtual and synchronous panel was organized with expert nurses in clinical simulation and HAI prevention, conducted via the Google Meet platform. During this stage, the experts assessed the objectives (purpose, goals, or intended outcomes) of the computer-based simulation, its structure/presentation (organization, framework, strategy, coherence, and adequacy), and its relevance (significance, impact, motivation, and engagement). Additionally, they were able to provide comments, critiques, and suggestions for modifications during the panel, all of which we recorded and subsequently analyzed in light of the selected theoretical framework.

## RESULTS

The process of transforming realistic clinical simulation scenarios into computer-based simulations required rigorous work, as prescribed by the adopted methods to ensure the quality of the developed artifact. In addition to the specific context of this study—teaching best practices for BSI prevention related to PVCs to nursing students and professionals—special consideration was given to a validated hybrid realistic simulation scenario previously assessed by subject matter experts^(3)^. The objective was to expand the reach of this simulation by transitioning it to a virtual environment (the identified problem) through the development of an artifact (a 3D virtual environment simulator).

### From conceptualization to computational representation

The design and creation of the artifact (computer-based simulation scenario) were carried out through processes of appropriation, repositioning, refocusing, and computational representation of key event elements.

The initial phase involved efforts to thoroughly assimilate the foundational realistic scenario to enable its transition to a virtual environment. To achieve this, we adopted a scene-oriented scenario description strategy, which included detailing the general storyline, identifying control points in its progression, and establishing breakpoints to guide the coding process in the software^(9)^.

Refocusing the learning objectives became necessary since, in a virtual environment, it would not be possible to perform practical technical skills associated with best practices for BSI prevention (such as hand hygiene with soap and water or alcohol-based hand rub) or specific nursing procedures (such as removal of a non-functional PVC and insertion of a new catheter) as originally planned in the realistic simulation scenario^(3)^.

In this version of the simulation, the focus on non-technical competencies, such as clinical reasoning and decision-making, rather than technical skills, was mitigated by combining it with other simulation modalities^(10)^. Furthermore, the advancement toward more immersive simulations incorporating virtual reality has recently been recognized as a viable alternative for providing more comprehensive nursing care learning experiences, as it enables repetition and deliberate practice in a controlled and safe environment^(6)^.

Anticipating the future standardization of a scenario description model for simulation, we established that each scene would include the following elements: a goal, the required non-technical skills, and the concrete operations needed to achieve that goal. For example, Scene 1 has the goal of “identifying the need for basic hand hygiene and performing it,” in which the participant, based on scene analysis and the clinical case circumstances (clinical reasoning skill), must decide to guide the nurse avatar to the sink and activate it.

As the scenes were structured according to the established standards, they were subsequently converted into programming language and implemented on the Unity 3D computational platform. During deliberative meetings, a multidisciplinary team continuously analyzed and refined multiple versions of the represented or implemented scenes.

The virtual simulation environment was modeled using a template (i.e., asset) freely available in the Unity 3D environment component repository (https://assetstore.unity.com/3d/environments). The two modeled environments included a nursing station (where the scenario execution begins) and a patient room (Figure 2). These environments were independent yet accessible via a door.

Although the template used already included physically realistic elements compatible with a hospital ward, such as patient beds and a hand hygiene sink, several additional elements had to be implemented to ensure compliance with the relevant regulations. Additionally, the team created specific objects for the scenario, such as the nurse call system used by the patient to request assistance from the nursing team in case of need or urgency.

The team also modeled two avatars: a newly arrived nurse on duty, representing the learner participant, and a patient using the nurse call system due to acute pain at the site of a saline-locked PVC. It is important to highlight that this problem-based scenario is recurrent, as it is part of the daily routine of many nursing professionals and students. Perhaps due to its common occurrence, implementing BSI prevention and control measures is still frequently overlooked.

Although the templates were sourced from freely accessible online repositories, several modifications were necessary to align them with the clinical case scenario, such as removing the patient avatar’s shoes (since they were lying in bed) and modeling a bedsheet to cover them. As recommended in the literature, these avatar adaptations were designed to enhance the alignment between the human forms represented in the scenario and the intended learning objectives of the simulation^(6)^.

### Interaction design choices

Regarding the interaction mode within the application, we chose the Point and Click style, which consists of interactions using only a mouse or other pointing devices (e.g., joystick, touchscreen, etc.). In this system, all actions performed by the participant—from moving the nurse avatar through the scenario to interacting with objects and the virtual patient—are executed through clicks. This interaction mode is simple and easy to learn, even for those unfamiliar with computers or video games.

**Figure 3:**
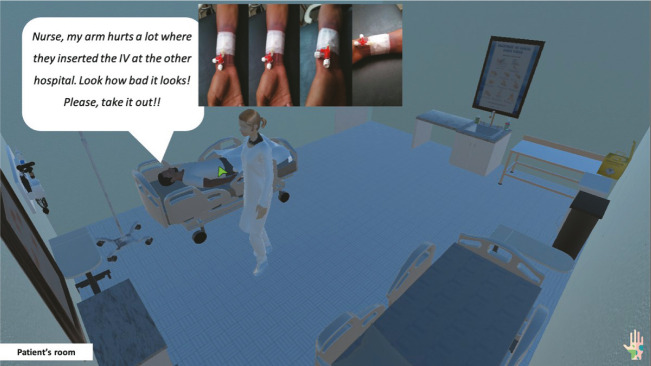
Screenshot of the interaction with the patient avatar, who reports a complaint

Each virtual environment offers different Point and Click interaction possibilities. In the nursing station environment, the participant can move the nurse avatar, identify interactive objects (which change the pointer color upon hovering), and access them. The same interaction model is implemented in the patient’s room, with the difference that, in this setting, the participant can also interact with the patient avatar (by clicking on it) to obtain information, such as the patient’s current complaint (Figure 3).

**Figure 4:**
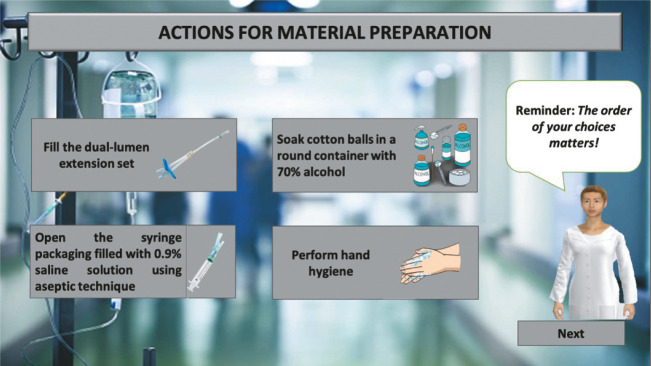
Screenshot of the Quest, displaying the steps for preparing materials for the required nursing procedure

Additionally, Quests (challenges/missions) were incorporated, appearing at key moments when participants must make decisions to progress in the simulation. For example, one Quest involves selecting the necessary steps to prepare materials for PVC insertion (Figure 4).

Both the Point and Click interactions and those presented through Quests can be triggered by the participant at their discretion and at any time they deem appropriate, regardless of whether the chosen action is correct or not. This freedom of choice was the approach adopted by the development team to replicate the non-linearity of real-world clinical practice in the virtual simulation. By allowing multiple action possibilities with corresponding consequences, the simulation avoids predefined prerequisites, fixed sequences, or a rigid flow of actions.

An example of this flexibility is hand hygiene. Although it is necessary at various points throughout the simulation, it was intentionally programmed as an optional action to encourage decision-making by the participant. The nurse avatar’s hands become visibly soiled when touching surfaces or the patient. Additionally, an icon in the lower right corner of the screen continuously indicates the current hygiene status, signaling whether the avatar’s hands are clean or dirty.

Since the technological choices made for this virtual simulation prevent the execution of certain technical procedures relevant to the case, instructional videos were incorporated to demonstrate step-by-step processes. Combining different digital learning resources, such as videos and podcasts, has been widely adopted as a complementary strategy to enhance educational outcomes.

At the end of the simulation, a report is generated detailing all actions performed chronologically. This report is intended to help participants self-assess their performance, encouraging reflection on their decisions and providing feedback on their experience with the virtual simulation. Incorporating this strategy is an effective tool for developing clinical reasoning in nursing students and professionals.

### Interface between implementation and future developments: evaluation through the prototype

Considering the importance of evaluating the artifact, the validity of the theoretical foundations used in its development, and its effectiveness in solving the identified problem^(7-9)^, a low-fidelity prototype was created using Microsoft PowerPoint. This prototype represented the system’s main screens in a two-dimensional format, with interactions simulated through clickable buttons. Additionally, it served as a didactic tool to illustrate the underlying organizational logic and content creation process within the simulation. The screen representations were adapted from adjusted screenshots of the three-dimensional version.

Thus, the prototype was presented in four parts: (1) briefing, (2) clinical case report, (3) clinical progression, and (4) feedback report.

In the briefing, preparatory information for the simulation was provided, including the simulation objectives, chronological organization, and descriptions of the environments and their components, the participants (the nurse avatar and the patient avatar), and the proposed interaction methods (Point and Click style and Quests).

Next, the clinical case report—which involved a low level of uncertainty—was presented, allowing the participant to practice clinical reasoning and decision-making while assisting a patient needing peripheral venous catheter (PVC) replacement. The reproduced case was entirely based on the validated realistic simulation scenario assessed by subject matter experts^(3)^.

The clinical progression was represented through a sequence of screens, illustrating the flow of scenes, the avatar’s movement within the two environments, the transition between spaces, and the required actions for task completion. Finally, the layout of the feedback report that the participant would receive at the end of the simulation was displayed.

For the evaluation, seven experts were invited, but only three agreed to participate in the virtual synchronous meeting. They assessed the computer-based simulation positively, recognizing its quality, theoretical validity, and ability to address the intended problem. Additionally, they suggested design modifications (aesthetic and grammatical), which were later analyzed and incorporated based on their relevance and alignment with the selected theoretical framework.

Since this is an iterative process^(9)^, it is important to clarify that the analyses and modifications resulting from the initial expert evaluation are part of the development cycle and are being used to inform decision-making regarding the continued refinement of the artifact.

## FINAL CONSIDERATIONS

The transition process described in this study resulted in the innovative development of a computer-based simulation designed to teach infection prevention measures for bloodstream infections related to peripheral venous catheters. This tool can potentially enhance autonomous, interactive, and engaging learning while optimizing material, physical, and financial resources.

Although a fully functional version of the artifact within the proposed three-dimensional environment is not yet available, the efforts undertaken have demonstrated both the potential benefits and challenges of this transition, particularly through the evaluation of a low-fidelity prototype.

Despite the advantages of this type of simulation, it is clear that it serves as a complementary technological tool rather than a replacement for laboratory-based training or real-world clinical experiences in both initial education and continuing professional development in nursing.

## Data Availability

The research data are available within the article.
