# Supplementary material for: Simulation-based training for teaching infection prevention: from realistic to virtual environments
Source: Rev Bras Enferm. 2025 Sep 1;78(Suppl 4):e20240288. doi: 10.1590/0034-7167-2024-0288 (PMC12419777; doi:10.1590/0034-7167-2024-0288)
Supplement: Translation

# Translation: Treinamento por simulação para o ensino de prevenção de infecções: do ambiente realístico ao virtual

**DOI:** 10.1590/0034-7167-2024-0288pt

**Authors:** Raissa Silva Souza, Dárlinton Barbosa Feres Carvalho, Adriana Maria da Silva Félix, Ana Angélica Lima Dias

**Affiliations:** I Universidade Federal de São João del-Rei. Divinópolis, Minas Gerais, Brazil; II Universidade de São Paulo. São Paulo, São Paulo, Brazil

**Keywords:** Tecnologia, Treinamento por Simulação, Raciocínio Clínico, Enfermagem, Infecções

## Abstract

**Objetivos:**

relatar o processo de transposição de uma simulação realística para uma baseada em computador sobre prevenção de infecção de corrente sanguínea.

**Métodos:**

este relato trata de uma solução baseada em tecnologias (simulador em ambiente virtual 3D) para um problema (ampliar o alcance de uma simulação realística já existente) contextualmente situado, utilizando a abordagem epistemológico-metodológica *Design Science Research*.

**Resultados:**

na plataforma computacional Unity 3D, desenvolveu-se um artefato (cenário de simulação baseada em computador), avaliado positivamente por painel de especialistas na área temática. Portanto, considera-se que o artefato tem qualidade, utiliza fundamentos teóricos válidos e pode resolver o problema ao qual se destina.

**Considerações Finais:**

o processo de transposição produziu uma simulação baseada em computador que favorece o aprendizado autônomo, lúdico e interativo. Contudo, é uma alternativa tecnológica complementar às práticas laboratoriais e às vivências clínicas.

## INTRODUÇÃO

A atual conjuntura educacional enfatiza os procedimentos de ensino, tornando-os tão importantes quanto os conteúdos de aprendizagem. Métodos e estratégias variados, com orientação teórica e pedagógica distintas, vêm sendo utilizados enquanto forma de fazer frente aos desafios oriundos dos espaços concretos das práticas^(1)^.

Pensando no campo da saúde, a preferência por metodologias que possibilitem a experimentação, a reflexão e a construção ativa do conhecimento pelos sujeitos é observada tanto em documentos oficiais como em publicações científicas^(1)^. Mais especificamente na área da enfermagem, a simulação clínica tem sido entendida como uma estratégia pedagógica capaz de criar condições para a aquisição e retenção de conhecimentos, assim como para o desenvolvimento de competências técnicas e não técnicas indispensáveis ao desempenho profissional, sejam elas psicomotoras, cognitivas ou atitudinais^(2)^.

No contexto do ensino de tópicos das boas práticas de prevenção das infecções relacionadas à assistência à saúde (IRAS), já existem na literatura^(3,4)^ cenários clínicos de simulação realística que podem levar estudantes e profissionais de saúde a revisar e a ressignificar suas práticas, qualificando-as e tornando-as mais efetivas e seguras. Entretanto, a aplicação desses cenários em diferentes espaços tem sido limitada por inadequação ambiental, escassez de recursos em diferentes ordens^(4)^ e insuficiência na qualificação de pessoal. Desse modo, o deslocamento para o domínio de soluções digitais, com uma virtualização da simulação realística, mostra-se uma alternativa promissora para superar as limitações observadas.

A simulação baseada em computador é um recurso pedagógico na qual se empregam tecnologias computacionais para criar ambientes virtuais de aprendizado interativos. Nesses ambientes, o participante procede à assistência requerida na situação proposta em sua totalidade — experienciando desde o recrutamento dos processos mentais necessários à reflexão sobre a situação até a escolha e execução das intervenções propriamente ditas — em um ambiente controlado e seguro para o participante e paciente^(5)^.

Dentre as potencialidades de uso da simulação baseada em computador para o ensino, encontram-se redução nos custos, baixo risco e níveis de ansiedade menores, possibilidade de treinamento em larga escala, reprodutibilidade, dispensa de espaços físicos e de recursos materiais ou humanos qualificados, dentre outros^(6)^. Além disso, é uma estratégia que se mostra eficaz no ensino de enfermagem, já que aprimora habilidades de pensamento crítico e de raciocínio clínico do estudante/profissional para que ele possa oferecer atendimento seguro aos pacientes, fazendo inferências precisas e tomando decisões baseadas em evidências. Não obstante, vale ressaltar que, para alcançar essas potencialidades, são necessários a pesquisa e o desenvolvimento tecnológico voltados para a criação de inovações adequadas aos propósitos almejados.

## OBJETIVOS

Relatar o processo de transposição de uma simulação realística para uma baseada em computador sobre prevenção das infecções de corrente sanguínea (ICS).

## MÉTODOS

Trata-se de relato sobre o desenvolvimento de uma solução baseada em tecnologias (simulador em ambiente virtual 3D) para um problema (ampliar o alcance de uma simulação realística já existente), utilizando a abordagem epistemológico-metodológica *Design Science Research* (DSR). Esse referencial consiste em um paradigma de desenvolvimento de solução embasada em tecnologias, criando artefatos para solução de problemas relevantes, complexos e contextualizados^(7)^. Para isso, o DSR propõe tanto a investigação científica rigorosa acerca do processo de criação de artefatos quanto a avaliação da adequação e da pertinência destes na resolução de problemas^(7)^.

Os elementos centrais do arcabouço metodológico empregado neste trabalho, conforme preconizado pelo DSR, encontram-se representados na Figura 1, que contém um modelo-síntese inspirado no trabalho de Pimentel, Filippo e Santoro^(7)^.

**Figura 1:** Arcabouço metodológico considerado neste relato

O contexto deste trabalho consiste em ensinar a estudantes e profissionais de enfermagem as boas práticas de prevenção de ICS relacionadas a cateteres venosos periféricos (CVP). Esse público tem disponível um cenário validado por especialistas das áreas temáticas para simulação realística híbrida, baseada em paciente simulado e em simulador de baixa fidelidade para injeções intravenosas^(7)^. Uma maneira de ampliar o alcance dessa simulação seria transpô-la para o ambiente virtual (o problema) por meio da proposição de um artefato (simulador em ambiente virtual 3D).

A condução do trabalho respeitou as seguintes etapas^(8)^: 1) conscientização – compreendeu-se e definiu-se o problema a ser solucionado, conforme exposto na Figura 1; 2) sugestão – foram elencadas as possíveis alternativas de artefato capazes de solucionar o problema e foi escolhida a que seria desenvolvida (simulador em ambiente virtual tridimensional); 3) desenvolvimento – correspondeu ao processo de construção do artefato em si; 4) avaliação iterativa – enfermeiras especialistas na área temática avaliaram o artefato, as teorias envolvidas em sua criação e o seu comportamento no ambiente para o qual foi projetado em relação às soluções pretendidas; 5) conclusão – consistiu na escrita do presente manuscrito para a formalização geral do processo e preparação para a comunicação; e 6) comunicação.

Entendendo a importância do desenvolvimento iterativo de artefatos tecnológicos cujo domínio da aplicação é complexo^(9)^, construiu-se um protótipo de baixa fidelidade para a etapa de avaliação. A finalidade foi demonstrar as diversas decisões de design tomadas pelos desenvolvedores e apresentar o desempenho alcançado como elemento último para se comprovar a adequação do artefato.

Considerando que a utilização de protótipos de baixa fidelidade em processos de avaliação de tecnologias é ainda incomum na área da enfermagem, optou-se por criar um painel virtual e síncrono com enfermeiros especialistas experientes nas áreas de simulação clínica e IRAS, via plataforma eletrônica Google Meet. Nessa etapa, os especialistas apreciaram os objetivos (propósitos, metas ou finalidades) da simulação computacional, sua estrutura/apresentação (organização, estrutura, estratégia, coerência e suficiência) e sua relevância (significância, impacto, motivação e interesse). Além disso, puderam comentar, fazer críticas ou sugerir modificações durante o painel, sendo todas registradas e, posteriormente, analisadas à luz do referencial teórico selecionado.

## RESULTADOS

O processo de transformar cenários clínicos de simulação realística naqueles de simulação baseada em computador requereu trabalho rigoroso, conforme preconizado pelos métodos adotados para garantir a qualidade do artefato concebido. Além de características do contexto deste trabalho de ensino das boas práticas de prevenção de ICS relacionadas a CVP a estudantes e profissionais de enfermagem, vale mencionar que foi considerado de forma especial um cenário para simulação realística híbrida validado por especialistas das áreas temáticas^(3)^. O intento foi ampliar o alcance dessa simulação com sua transposição para o ambiente virtual (o problema) mediante a proposição de um artefato (simulador em ambiente virtual 3D).

### Da apropriação à representação via artefato computacional

A concepção e a criação do artefato deste estudo (cenário de simulação baseada em computador) deram-se por meio de movimentos de apropriação, recolocação, refocalização e representação computacional dos elementos essenciais dos eventos.

O processo inicial envolveu esforços para assimilar, em profundidade, a proposta contida no cenário realístico de base a fim de transpô-lo para o ambiente virtual. Para tanto, adotou-se a estratégia da descrição de cenários orientados à cena, tendo feito o detalhamento do cenário (enredo geral), a identificação de pontos de controle na progressão deste e o estabelecimento dos pontos de ruptura, com vistas à codificação no *software* ^(9)^.

Decorreu desse movimento, a necessidade de se refocalizarem os objetivos de aprendizagem, uma vez que, no ambiente virtual, não seria possível o desempenho prático de habilidades técnicas pertinentes às boas práticas de prevenção ICS (como higiene simples das mãos e fricção com preparação alcoólica) e de procedimentos de enfermagem específicos (como a retirada de CVP não funcionante e inserção de um novo cateter) previstos no cenário realístico^(3)^.

Nesta versão da simulação, o foco no desempenho de competências não técnicas (como o raciocínio clínico e a tomada de decisão) em detrimento das técnicas propriamente ditas tem sido mitigado por sua combinação com outras modalidades de simulação^(10)^. Além disso, o avanço para simulações mais imersivas, com uso da realidade virtual, tem sido mais recentemente uma alternativa reconhecida como capaz de viabilizar experiências de aprendizagem mais completas da assistência de enfermagem, por permitir a repetição e a prática deliberada em ambiente seguro e controlado^(6)^.

Vislumbrando a utilização futura de um modelo para descrição de cenários para simulação, padronizou-se que cada cena teria os seguintes itens: uma meta, as habilidades não técnicas e as operações concretas a serem desempenhadas para o alcance dessa meta. Por exemplo: a Cena 1 tem por meta “identificar a necessidade da realização da higiene simples das mãos e realizá-la”, sendo que o participante deverá, mediante a análise da cena e das circunstâncias apresentadas no caso clínico (habilidade de raciocínio clínico), tomar a decisão de guiar o avatar da enfermeira até o lavatório e acioná-lo.

À medida que as cenas eram representadas nos padrões estabelecidos, realizava-se tanto sua conversão para a linguagem de programação quanto implementação na plataforma computacional Unity 3D. Em reuniões deliberativas, uma equipe multiprofissional foi analisando e retificando inúmeras versões das cenas representadas ou implementadas.

O ambiente da simulação virtual foi modelado com base em um molde (i.e., *asset*), disponível gratuitamente no repositório de componentes de ambiente tridimensional da Unity (https://assetstore.unity.com/3d/environments). Os dois ambientes representados foram um posto de enfermagem (Figura 2) (ambiente em que a execução do cenário é iniciada) e um quarto de paciente (Figura 2). Eles eram independentes e acessíveis por meio de uma porta.

**Figura 2:** Representação do posto de enfermagem no cenário clínico (à esquerda) e do quarto do paciente (à direita) no cenário clínico, modelados na plataforma computacional *Unity 3D*

Apesar de o molde utilizado já conter elementos de fidelidade física compatíveis com os de uma unidade de internação hospitalar, como leitos para pacientes e lavatório para a higienização das mãos, foi preciso implementar uma série de outros elementos para tornar o ambiente apropriado conforme a regulamentação pertinente. Além disso, também foram criados objetos específicos do cenário, como o sistema de chamada de enfermagem utilizado pelo paciente para acionar a equipe de enfermagem em um momento de necessidade ou urgência.

A equipe também modelou dois avatares: uma enfermeira recém-chegada ao plantão, que representa o participante aprendiz; e um paciente que está chamando a enfermagem pelo sistema de chamada por sentir uma dor aguda no local onde existe um CVP salinizado. Ressalta-se que a situação-problema retratada nesse caso é recorrente, pois faz parte do cotidiano de muitos profissionais e estudantes de enfermagem. Talvez, por mostrar-se uma situação habitual, a aplicação de medidas de prevenção e de controle de ICS ainda sejam negligenciadas.

Apesar de os moldes terem sido encontrados na modalidade de acesso livre na internet, várias adaptações foram necessárias para torná-los compatíveis com o caso clínico proposto, como retirar os sapatos do avatar paciente (deitado no leito) e modelar o lençol que o cobriria. Como recomendado na literatura, as adaptações realizadas nos avatares buscaram favorecer o alinhamento entre as formas humanas retratadas no cenário e os objetivos de aprendizagem propostos com a simulação^(6)^.

### As escolhas em torno da interação

Em se tratando da forma de interação na aplicação, optou-se pelo estilo *Point and Click*, que consiste apenas em interações com um mouse ou com outros dispositivos apontadores (p.ex., *joystick*, tela sensível ao toque etc.). Dessa forma, todas as ações executadas pelo participante, desde andar com o avatar pelo cenário até interagir com os objetos e com o paciente virtual, são realizadas por meio de cliques. É um modo de interação simples e de fácil aprendizado mesmo para aqueles não familiarizados com computadores ou jogos eletrônicos.

Em cada ambiente virtual, há diferentes possibilidades de interação no estilo *Point and Click.* No ambiente que representa o posto de enfermagem, é possível ao participante movimentar o avatar da enfermeira pelo espaço, identificar objetos interativos (por meio da mudança na cor do ponteiro) e acessá-los. A mesma proposta de interação está implementada no quarto do paciente. A diferença é que, nesse ambiente, o participante poderá também interagir com o avatar do paciente (clicando com o mouse*)* para dele obter informações, por exemplo, sobre sua queixa atual (Figura 3).

**Figura 3:** Captura da tela da interação com o avatar do paciente, que refere queixa

Ainda, ressalta-se a utilização de *Quests* (desafios/missões), apresentadas ao participante em momentos nos quais ele precisaria tomar decisões para avançar na simulação. Por exemplo, havia uma *Quest* para a seleção dos passos necessários ao preparo de materiais para a inserção de CVP (Figura 4).

**Figura 4:** Captura da tela da *Quest* contendo as etapas de preparo dos materiais para a realização do procedimento de enfermagem requerido

Tanto as interações por meio do estilo *Point and Click* quanto aquelas mediante *Quests* podem ser acionadas pelo participante da forma como ele julgar apropriada e quando considerar oportuno, independentemente de ser adequada ou não. Essa liberdade de escolha foi a maneira encontrada pela equipe de representar a não linearidade do mundo real na simulação virtual: gerar várias possibilidades de ação com as devidas consequências, sem pré-requisitos, ordem fixa ou um fluxo pré-estabelecido e fixo para as ações.

Um exemplo é a higienização das mãos. Apesar de necessária em vários momentos ao longo da simulação, foi programada para ser uma ação intencionalmente opcional, de modo a oportunizar a tomada de decisão pelo participante. As mãos do avatar sujam-se ao tocar em superfícies e/ou no paciente. De toda forma, é uma condição reforçada por meio de ícone, apresentado no canto inferior direito da tela, indicando se as mãos estão limpas ou sujas.

Como as alternativas tecnológicas escolhidas para essa simulação virtual inviabilizam procedimentos técnicos pertinentes ao caso, optou-se por inserir vídeos nos quais o passo a passo é apresentado. A combinação de diferentes recursos digitais de aprendizagem (vídeos, podcasts etc.) tem sido muito utilizada de forma complementar.

Ao término da simulação, um relatório descrevendo as ações desempenhadas na sequência cronológica é entregue ao participante. Espera-se que ele possa autoavaliar-se ao refletir sobre o relatório, obtendo um feedback como resultado da experiência com a simulação virtual. A incorporação de tal estratégia é um elemento efetivo para desenvolver o raciocínio clínico nos estudantes e profissionais de enfermagem.

### Interface entre o implementado e o porvir: avaliação por meio do protótipo

Considerando a importância da avaliação do artefato, da validade dos fundamentos utilizados em sua construção e da sua capacidade de resolver o problema^(7-9)^, construiu-se um protótipo de baixa fidelidade no *software* de criação de apresentações Microsoft PowerPoint. Ali foram representadas as telas principais do sistema bidimensionalmente e foram simuladas as interações mediante botões. Usando o protótipo, também foi possível demonstrar didaticamente os fundamentos e a lógica subjacente ao processo de organização, bem como a criação dos conteúdos representados na simulação. Vale dizer que as representações das telas foram obtidas por meio de ajustamentos de capturas de telas da versão tridimensional.

Portanto, o protótipo foi reapresentado em quatro partes: 1) *briefing*; 2) relato do caso clínico; 3) progressão clínica; e 4) relatório de feedback.

No *briefing,* foram apresentadas as informações preparatórias para a simulação, tais como objetivo da simulação, organização cronológica e descrição dos ambientes e de seus elementos componentes, dos participantes (o avatar que representa a enfermeira e o que representa o paciente) e das formas de interação propostas (estilo *Point and Click* e *Quests*).

Em seguida, exibe-se o relato do caso clínico — de baixo nível de incerteza — no qual o participante exercita o processo de raciocínio clínico e a tomada de decisão na assistência ao paciente com necessidade de substituição de CVP. O caso reproduzido baseou-se integralmente na versão da simulação realística validada por especialistas^(3)^.

A progressão clínica foi representada em uma sequência de telas que reproduziu o encadeamento das cenas, a circulação do avatar nos dois ambientes, seu trânsito entre os ambientes e a forma como as ações devem ser realizadas em cada um. Por fim, expõe-se o layout do feedback que o participante recebe ao final da simulação.

Para a avaliação, foram convidados sete especialistas, porém apenas três se dispuseram a participar da reunião virtual e síncrona. Eles consideraram que a simulação baseada em computador tem qualidade, utiliza fundamentos teóricos válidos e mostra-se capaz de resolver o problema ao qual se destina. Além disso, recomendaram mudanças no design (estéticas e gramaticais), que foram posteriormente analisadas e acatadas, considerando sua pertinência e convergência com o referencial selecionado.

Como se trata de um processo iterativo^(9)^, é importante esclarecer que as análises e as transformações advindas da avaliação inicial dos especialistas fazem parte do percurso e estão sendo utilizadas como subsídio para a tomada de decisão sobre a continuidade do desenvolvimento do artefato.

## CONSIDERAÇÕES FINAIS

O processo de transposição relatado culminou na criação inventiva de uma simulação baseada em computador voltada ao ensino das medidas de prevenção das infecções de corrente sanguínea relacionadas a cateteres venosos periféricos. Essa ferramenta tem potencial de favorecer o aprendizado autônomo, lúdico e interativo, com otimização de recursos materiais, físicos e financeiros.

Apesar de ainda não haver uma versão funcional do artefato no ambiente tridimensional proposto, os esforços envidados permitiram demonstrar o potencial e as dificuldades de se realizar essa forma de transposição, obtida principalmente por meio da avaliação de um protótipo de baixa fidelidade.

A despeito das potencialidades dessa modalidade de simulação, fica claro tratar-se de uma alternativa tecnológica complementar, portanto não substitui as práticas laboratoriais nem as vivências clínicas conduzidas em ambientes reais de aprendizagem na formação inicial e continuada de profissionais de enfermagem.

## Data Availability

Os dados da pesquisa estão disponíveis no corpo do artigo.
